# Schaftoside Reduces Depression- and Anxiogenic-like Behaviors in Mice Depression Models

**DOI:** 10.3390/brainsci15030238

**Published:** 2025-02-24

**Authors:** Yue Hu, Yaoxue Gan, Jia Lei, Jinhui Cai, Yecheng Zhou, Hao Chen, Qian Zhang, Yan Shi

**Affiliations:** School of Medical Technology and Translational Medicine, Hunan Normal University, Changsha 410006, China; 202220193561@hunnu.edu.cn (Y.H.); kwx0130@hunnu.edu.cn (Y.G.); 202230193042@hunnu.edu.cn (J.L.); 202230191090@hunnu.edu.cn (J.C.); 202230191031@hunnu.edu.cn (Y.Z.); 202230191048@hunnu.edu.cn (H.C.); 202230191039@hunnu.edu.cn (Q.Z.)

**Keywords:** depression, anxiety, schaftoside, chronic unpredictable mild stress, inflammation

## Abstract

Background: Major depressive disorder is a common mental health issue characterized by persistently low mood and high morbidity and mortality. The major pathophysiology is neuroinflammation, as evidenced by elevated cytokine levels. Patients often fail to achieve full remission with the use of currently available antidepressants, prompting the search for new treatment options. Schaftoside (SS), a flavonoid found in traditional Chinese herbs, has both antioxidant and anti-inflammatory properties. However, its antidepressant effects are poorly understood. Methods: Male C57BL/6 mice underwent chronic unpredictable mild stress (CUMS) and lipopolysaccharide (LPS) treatment to induce depression- and anxiety-like behaviors. SS was administered at 40, 80, and 160 mg/kg for 28 days. The effect on depression-like behaviors was assessed using behavioral assays, and ELISA was used to measure pro-inflammatory cytokines in the serum and hippocampus. Results: SS significantly decreased immobility in the forced swim and tail suspension tests, increased sucrose preference in the sucrose preference test, and reduced feeding latency in the novelty-suppressed feeding test. These findings indicate improved depression and anxiety-like behaviors. ELISA showed that SS lowered interleukin-1 beta (IL-1β), IL-6, and tumor necrosis factor-alpha levels in the serum and hippocampus of CUMS mice. Conclusions: Our study indicates that SS has antidepressant and anxiolytic effects, possibly through neuroinflammatory processes, making it a promising therapeutic candidate for depression, and thus deserves further investigation into its mechanisms and clinical efficacy.

## 1. Introduction

Major depressive disorder (MDD) is the third leading cause of non-fatal health complications [1]. A 2020 study revealed a significant increase (27.6%) in the incidence of new MDD cases, affecting more than 53 million people [2]. MDD is associated with a significant mortality rate, reaching up to 6% primarily following suicide [3]. Typically, it is characterized by pervasive low mood and depressive behaviors including disturbances in sleep, memory, and attention as well as possible physical manifestations such as reduced energy, motivation, sexual desire, and appetite [4].

The etiology of depression is primarily attributed to various factors including prolonged stress exposure, sex disparities, behavioral patterns, and additional elements [5]. Recently, the pathogenesis of MDD has involved advancements in monoamine neurotransmitters, gut–brain axis, hypothalamic–pituitary–adrenal axis, and neuroinflammatory responses [6,7,8]. Animal and microglia studies have indicated that depression is associated with neuroinflammation [9,10] including sterile inflammation, glial cell activation, and elevated levels of interleukin-6 (IL-6), IL-10, IL-1β, and tumor necrosis factor-alpha (TNF-α) alongside reduced gamma interferon (IFN-γ) concentrations [11]. These results suggest that patients with depression exhibit a chronic, low-grade inflammatory state with immune dysregulation. In 1991, Smith et al. first identified that macrophage-induced inflammation might be crucial for the onset of depression [12]. Subsequently, studies have demonstrated a significant relationship between inflammatory response and depression. Concurrently, investigations on the relationship between antidepressants and inflammatory factors are rapidly increasing [13,14]. Numerous antidepressant drugs have been developed; however, notably, a considerable number of patients do not achieve complete recovery following sufficient treatment with these medications [15,16]. Studies have revealed a notable decrease in TNF-α levels among responders to antidepressant treatment, contrasting with the results in non-responders [14]. This suggests a significant association between alterations in the levels of certain inflammatory factors and the efficacy of antidepressants. Globally, the effects of inflammatory responses on the development of depression based on the cytokine hypothesis are being investigated. However, most existing studies focused on phenomenological associations and the outcomes of specific molecular interventions. Therefore, it is imperative to investigate the pathophysiological mechanisms underlying depression and explore novel therapeutic approaches for depression.

Recently, many traditional Chinese medicines and their natural molecules have shown promising anti-neuroinflammatory effects [17,18,19,20]. These findings indicate that traditional Chinese medicine and its constituents hold significant potential and a promising application in the treatment of neuroinflammation. Flavonols, a subclass of flavonoids commonly present in the human diet, are emerging as potentially effective and safe treatment options for depression [18] due primarily to their notable antioxidant and anti-inflammatory properties. Schaftoside (SS) is a flavonoid present in several traditional Chinese medicinal herbs including Dendrobium nobile, Eleusine indica, Glycyrrhiza uralensis, and Lysimachia christinae [21]. Previous studies have shown that SS exhibits several pharmacological activities including autophagy regulation [22], antioxidative stress [23], and anti-inflammatory activity [23,24]. Inflammation, including the upregulation of IL-6, IL-1β, and the nuclear factor kappa-light-chain enhancer of activated B cells (NF-κB), was downregulated following pretreatment with SS in a model of pentylenetetrazol-induced epileptic seizures in zebrafish [24].

Despite its range of pharmacological effects, the antidepressant effects of SS have not been reported. Therefore, due to the relationship between depression and neuroinflammation, we speculated that SS may exert its antidepressant effects by improving neuroinflammation. Our findings showed that SS effectively alleviated depression- and anxiety-like behaviors in mice subjected to lipopolysaccharide (LPS) treatment and chronic unpredictable mild stress (CUMS). Moreover, the reduction in pro-inflammatory cytokine levels by SS may underlie its effects.

## 2. Material and Methods

### 2.1. Animals

Male C57BL/6 mice, 4 weeks old, weighing 15–20 g, were housed in a controlled environment at a constant temperature of 25 °C with a 12-h light/dark cycle. The mice had free access to food and tap water throughout the study. All mice were housed individually in separate cages throughout the entire experimental period. All experiments were conducted during the dark phase and were approved by the Local Committee on Animal Care, Use, and Protection of Hunan Normal University.

### 2.2. Reagents and Drugs

SS powder (1 mg, MCE, Cat. No.: HY-N0703, CAS No. 51938–32-0, MedChemExpress, Shanghai, China) was dissolved in dimethyl sulfoxide (DMSO; Sigma, St. Louis, MO, USA). SS was initially dissolved in fresh DMSO to prepare a concentrated stock solution. For in vivo administration, the stock solution was further diluted with a vehicle mixture containing 40% polyethylene glycol 300, 5% Tween 80, and 45% saline. The DMSO content in the final solution administered to the animals was maintained at 10% (*v*/*v*), a concentration widely reported as well-tolerated in rodent studies, to avoid solvent-related toxicity. LPS (L2880; Sigma, MO, US) and fluoxetine (Flx, T0450; TargetMol, Shanghai, China) were dissolved in physiological saline.

### 2.3. Experimental Design

#### 2.3.1. Experiment 1: Effects of SS Treatment on Locomotor Activity in Open Field Test and Immobility Time in the Forced Swim Test (FST) in Mice for 28 Days

In this experiment, the mice were habituated for 7 days and randomly divided into five groups: SS 40 mg/kg (*n* = 10), SS 80 mg/kg (*n* = 9), SS 160 mg/kg (*n* = 10), fluoxetine 10 mg/kg (*n* = 10), and vehicle (*n* = 10). Behavioral tests were conducted following the final oral gavage with a 24-h interval, after 28 consecutive days of daily oral administration of either the vehicle or SS to the mice. Fluoxetine was used as a positive control.

#### 2.3.2. Experiment 2: Effects of SS Treatment on Depression- and Anxiety-like Behaviors Induced by LPS for 28 Days

In Experiment 2, a mouse model of depression induced by LPS was used to examine behavioral responses to SS. A distinct group of mice was acclimated for 7 days and randomly assigned into four groups: vehicle + saline (*n* = 10), vehicle + LPS (*n* = 10), SS 80 mg/kg + LPS (*n* = 11), and SS 160 mg/kg + LPS (*n* = 11). The mice received vehicle or SS (80 and 160 mg/kg) treatment via oral gavage once daily for 28 consecutive days. On Days 29 and 30, the mice were injected with LPS (2 mg/kg) or saline once daily. Behavioral tests were conducted following the final injection with a 24-h interval.

#### 2.3.3. Experiment 3: Effects of SS Treatment on Depression- and Anxiety-like Behaviors Induced by CUMS for 28 Days

Following a 7-day habituation period, different sets of mice were randomly assigned to four groups: vehicle (*n* = 10), CUMS + vehicle (*n* = 11), CUMS + SS (*n* = 11), and CUMS + Flu (*n* = 10). The mice were subjected to CUMS for 28 days or housed individually. Simultaneously, the mice were orally administered daily doses of either the vehicle or 80 mg/kg SS for 28 consecutive days. The mice were subjected to behavioral tests following the final oral gavage with a 24-h interval.

### 2.4. CUMS

The CUMS procedure was performed as previously described. Briefly, male C57BL/6 mice were randomly assigned to either the control or CUMS model group. Animals in the control group were housed under standard conditions. In contrast, the CUMS model group was subjected to different stressors daily for 5 weeks including fasting (24 h), water deprivation (24 h), cage tilting (45°, 24 h), empty or wet cage (24 h), random noise stimulation (6 h), restraint (30 min), horizontal shaking (5 min), and tail nip (1 min).

### 2.5. Behavioral Assays

#### 2.5.1. FST

Each mouse was individually placed in a circular glass container with a diameter and height of 20 and 30 cm, respectively, filled with room-temperature water reaching a depth of 20 cm. The forced swimming period of each mouse was monitored for 6 min. The duration of immobility, defined as the absence of escape behavior during the last 4 min of each test, was analyzed.

#### 2.5.2. Sucrose Preference Test (SPT)

A 24-h acclimation period was implemented before initiating the formal test to familiarize the animals with the 1% sucrose solution. During the experiment, the animals were deprived of water for 24 h. During this period, each cage was equipped with one bottle of 1% sucrose solution and another bottle of pure water for the mice to consume. The positions of the bottles were switched every 12 h to minimize potential bias. The sucrose solution and pure water were provided for a duration of 24 h. The sugar preference ratio was calculated using the following formula: sugar preference ratio (%) = [sugar intake/(sugar intake + pure water intake)] × 100%.

#### 2.5.3. Tail Suspension Test (TST)

During the experiment, the mice were suspended 20–25 cm above the floor using adhesive tape affixed to a point 1 cm from the tail tip. Mice behavior was recorded for 6 min using a video camera. The latency to give up struggling and the duration of immobility during the last 4 min of each trial were analyzed.

#### 2.5.4. Open Field Test (OFT)

The open field, a square enclosure measuring 50 × 50 × 40 cm on each side, was used for the OFT. Each mouse was placed in the field for free exploration for 5 min. A camera system was used to record the movement trajectory of each mouse. Afterward, data on the motion distance and frequency of entries into the central zone were analyzed using SuperMaze video analysis software (Shanghai XinRuan Information Technology Co. Ltd., Shanghai, China).

#### 2.5.5. Novelty-Suppressed Feeding Test (NSFT)

We conducted the NSFT to assess anxiety-like behaviors in the mice under novel conditions. The mice were subjected to a 24-h period of food deprivation before testing. During the 5-min test session, each mouse was placed in the corner of an open-field arena (50 × 50 × 40 cm) that had a 10 × 10 cm white paper sheet at the center with small food pellets. The latency to feed, defined as the time taken by the mouse to initiate feeding, was recorded. After the test session, the mice were returned to their home cages, and the total food consumption was measured for 5 min to account for the potential effects of food deprivation.

### 2.6. Enzyme-Linked Immunosorbent Assay (ELISA)

Frozen whole hippocampal tissues were lysed with a radio-immunoprecipitation assay buffer, homogenized, and centrifuged. The supernatants were collected and stored at −80 °C for subsequent analysis. Levels of pro-inflammatory cytokines (IL-1β, IL-6, and TNF-α) in the hippocampus were measured using a commercial ELISA kit (ABclonal, Wuhan, China), following the manufacturer’s protocol.

### 2.7. Statistical Analysis

The results were presented as the mean ± standard error of the mean (SEM). Statistical analyses were conducted using GraphPad Prism (version 9). A one-way or two-way analysis of variance was used, followed by Tukey’s post hoc test. Statistical significance was set at *p* < 0.05.

## 3. Results

### 3.1. A 28-Day Treatment with SS Yielded an Antidepressant Effect in the FST

**Figure 1:**
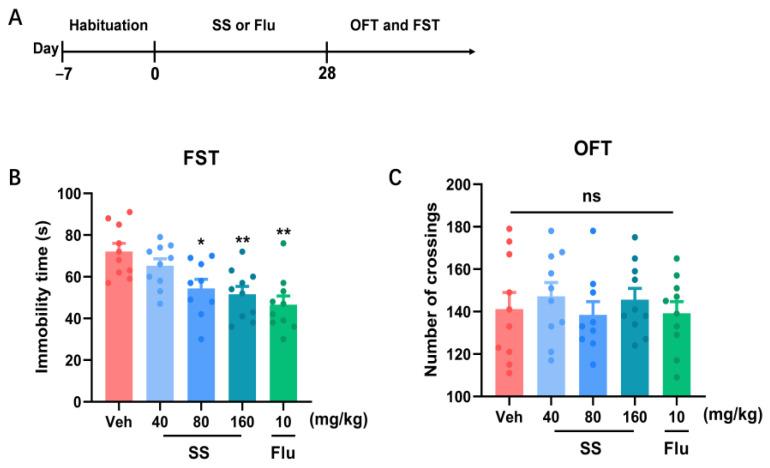
A 28-day treatment with SS yielded an antidepressant effect in the FST. (**A**) Timeline of the experiment. (**B**) Immobility time in FST. (**C**) Number of crossings in OFT. Data were expressed as the mean ± SEM (*n* = 9–10 per group). * *p* < 0.05, ** *p* < 0.01 vs. Veh group. ns means *p* > 0.05 no statistical difference.

We initially explored the antidepressant effects of various SS doses over 28 days (Figure 1A). In addition to the 40 mg/kg dose (*p* = 0.5487), intragastric administration of SS (80 mg/kg) [*p* = 0.0106], SS (160 mg/kg) [*p* = 0.0020], and Flu (10 mg/kg) [*p* = 0.0001] markedly reduced the immobility time (F_4,44_ = 6.982, *p* = 0.0001; Figure 1B) in the FST (Figure 1B) compared with the vehicle group. This indicated that SS yielded a depression-alleviating effect in the FST. In the OFT, no notable variations were observed in the number of crossings (F_4,44_ = 0.3691, *p* = 0.8293; Figure 1C) between the SS (40, 80, and 160 mg/kg) and Flu (80 and 160 mg/kg) groups relative to the vehicle group, indicating an unaffected motor function in the mice.

### 3.2. Prolonged Treatment of SS Improved Depression- and Anxiety-like Behaviors Caused by LPS

**Figure 2:**
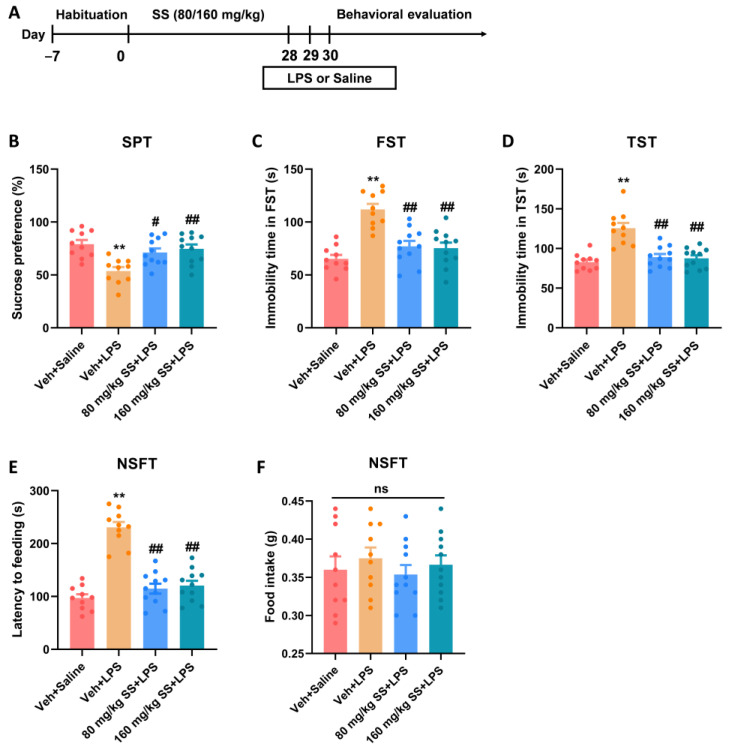
Prolonged treatment of SS improved depression- and anxiety-like behaviors caused by LPS. (**A**) Timeline of the experiment. (**B**) Sucrose preference of SPT. (**C**) Immobility time in FST. (**D**) Immobility time in TST. (**E**) Latency to feeding in NSFT. (**F**) Food stake in NSFT. Data were expressed as the mean ± SEM (*n* = 10–11 per group). ** *p* < 0.01 vs. Veh + Saline group; ^#^ *p* < 0.05, ^##^ *p* < 0.01 vs. Veh + LPS group; ns means *p* > 0.05 no statistical difference.

Due to the significant antidepressant effects of SS in the FST, further experiments were performed using 80 and 160 mg/kg SS. SS was administered to assess its effect on LPS-related depression- and anxiety-like behaviors in mice (Figure 2A). In the SPT (Figure 2B), mice in the 80 and 160 mg/kg SS + LPS groups showed considerably higher sucrose preference (F_3,38_ = 7.583, *p* = 0.0004) than that of mice in the LPS group, indicating a reduction in anhedonia. Furthermore, the 80 and 160 mg/kg SS + LPS groups exhibited considerably shorter immobility time in the FST (F_3,38_ = 16.46, *p* < 0.0001; Figure 2C) and TST (F_3,38_ = 17.64, *p* < 0.0001; Figure 2D) than in the LPS group. These results demonstrate that SS improves LPS-induced depression-like behaviors in mice. In the NSFT (Figure 2E,F), the 80 and 160 mg/kg SS + LPS groups demonstrated decreased latency to feed (F_3,38_ = 42.11, *p* < 0.0001) compared with the LPS group, whereas food intake (F_3,38_ = 0.4148, *p* = 0.7434) remained essentially unchanged, indicating that SS alleviated anxiety-like behaviors caused by LPS in the mice.

### 3.3. Prolonged Treatment of SS Improved Depression- and Anxiety-like Behaviors in CUMS Mice

**Figure 3:**
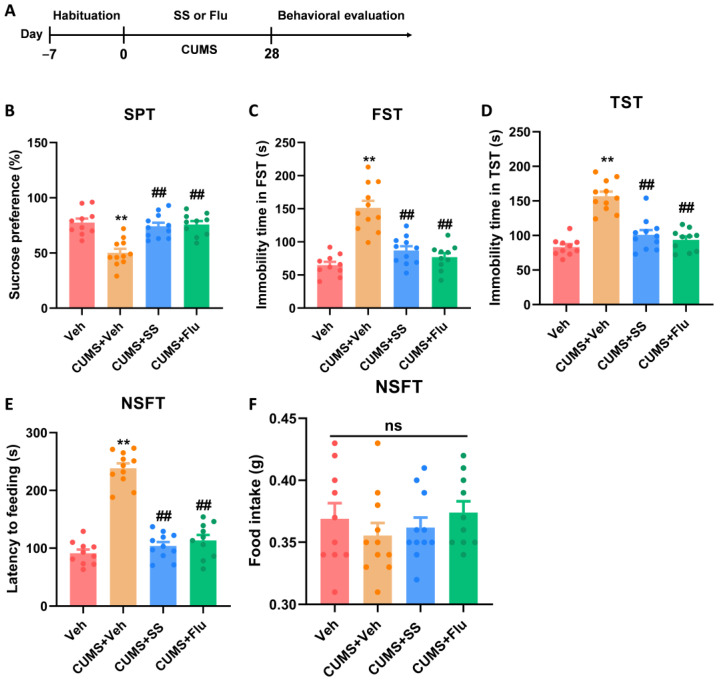
Prolonged treatment of SS improved depression- and anxiety-like behaviors in CUMS mice. (**A**) Timeline of the experiment. (**B**) Sucrose preference of SPT. (**C**) Immobility time in FST. (**D**) Immobility time in TST. (**E**) Latency to feeding in NSFT. (**F**) Food stake in NSFT. Data were expressed as the mean ± SEM (*n* = 10–11 per group). ** *p* < 0.01 vs. Veh group; ^##^ *p* < 0.01 vs. CUMS + Veh group; ns means *p* > 0.05 no statistical difference.

In the present study, we investigated whether SS could improve depression- and anxiety-like behaviors in mice using the CUMS model (Figure 3A). In the SPT (Figure 3B), mice in the SS + CUMS and Flu + CUMS groups showed considerably higher sucrose preference (F_3,38_ = 14.31, *p* < 0.0001) in contrast to those in the CUMS group, implying that SS and Flu improved anhedonia. In addition, the SS + CUMS and Flu + CUMS groups had significantly shorter immobility time in the FST (F_3,38_ = 26.45, *p* < 0.0001; Figure 3C) and TST (F_3,38_ = 32.10, *p* < 0.0001; Figure 3D) compared with the CUMS group. These findings show that SS and Flu improved the depression-like behaviors in mice using the CUMS model. Furthermore, in the NSFT (Figure 3E,F), the SS + CUMS and Flu + CUMS groups showed decreased latency to feed (F_3,38_ = 76.57, *p* < 0.0001) compared with the CUMS group; however, food intake (F_3,38_ = 0.6563, *p* = 0.5840) remained largely the same, indicating that SS alleviated anxiety-like behaviors in mice in the CUMS model.

### 3.4. Chronic Administration of SS Reduced Pro-Inflammatory Cytokine Concentrations in the Serum and Hippocampus of CUMS Mice

**Figure 4:**
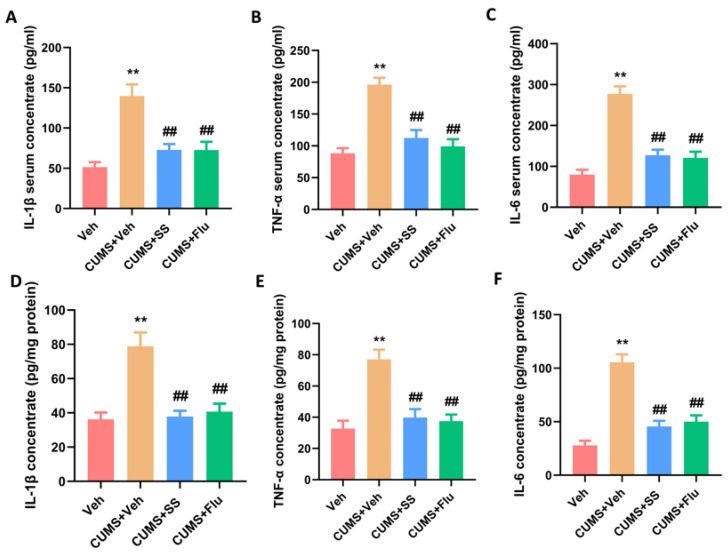
Chronic administration of SS reduced pro-inflammatory cytokine concentrations in the serum and hippocampus of CUMS mice. (**A**–**C**) ELISA of IL-1β, IL-6, and TNF-α in the serum of each group. (**D**–**F**) ELISA of IL-1β, IL-6, and TNF-α in hippocampus of each group. Data were expressed as the mean ± SEM. ** *p* < 0.01 vs. Veh group; ^##^ *p* < 0.01 vs. CUMS + Veh group; ns means *p* > 0.05 no statistical difference.

ELISA experiments were used to detect the IL-1β, IL-6, and TNF-α levels in the serum and hippocampus in mice. In the CUMS group, the IL-1β (F_3,20_ = 14.39, *p* < 0.0001), TNF-α (F_3,20_ = 20.92, *p* < 0.0001) and IL-6 (F_3,20_ = 33.17, *p* < 0.0001) levels in the serum were elevated, which were notably reduced following the administration of SS and Flu (Figure 4A–C). Furthermore, the hippocampal IL-1β (F_3,20_ = 14.41, *p* < 0.0001), IL-6 (F_3,20_ = 14.74, *p* < 0.0001), and TNF-α (F_3,20_ = 32.62, *p* < 0.0001) levels, elevated in the CUMS group, were markedly reduced in the SS + CUMS and Flu + CUMS groups (Figure 4D–F).

## 4. Discussion

Depression is a prevalent mental health condition and is closely associated with neuroinflammation. SS, a flavonoid extracted from various traditional Chinese medicinal herbs, possesses anti-inflammatory characteristics [25]. In the present study, SS ameliorated depression- and anxiety-like behaviors in mice treated with LPS and subjected to CUMS. Furthermore, the pro-inflammatory cytokine (IL-1β, IL-6, and TNF-α) levels in the hippocampus and serum of the CUMS model mice were reduced by SS. Our research demonstrates that SS could emerge as a viable treatment for depression.

Recent studies have demonstrated a relationship between inflammation and depression pathophysiology. Microglial activation has been detected in various animal depression models [26,27,28]. Activated microglia secrete reactive oxygen species, IL-1β, IL-6, and TNF-α [29], thereby actively promoting inflammatory signaling [30]. Patients with MDD exhibit elevated concentrations of inflammatory markers in their bloodstream [31,32]. Consequently, the inflammatory process is essential in the development of depression, and anti-inflammatory approaches are emerging as significant therapeutic strategies. Many drugs can treat depression due to their anti-inflammatory effects. For example, minocycline demonstrates its anti-inflammatory properties by preventing microglia activation, thereby inhibiting the production of IL-1β, IL-6, IL-2, TNF-α, and IFN-γ [33]. Minocycline has anti-inflammatory effects, protects neurons, and reduces oxidative stress [34]. Furthermore, cytokine inhibitors can improve depression by directly inhibiting inflammatory cytokines, consequently mitigating the detrimental effects of inflammation on the nervous system [35]. Cytokine inhibitors have demonstrated significant antidepressant effects in patients with depression and elevated inflammation [36].

LPS administration and chronic inflammation can stimulate the immune system to secrete inflammatory cytokines peripherally and in the brain [37,38]. These cytokines penetrate the blood–brain barrier to directly affect cerebral function or activate microglia, leading to neuronal atrophy in depression-related regions including the prefrontal cortex and hippocampus. In addition, LPS-induced inflammation disrupts the hypothalamic–pituitary–adrenal axis and alters neurotransmitter levels, contributing to the development of depression-like features [39]. In our study, both the LPS-treated and CUMS model mice exhibited depression- and anxiety-like behaviors. The IL-1β, IL-6, and TNF-α levels in the hippocampal region and blood serum of CUMS model mice were elevated.

Emerging evidence highlights the promising role of dietary flavonoids and polyphenols—ubiquitous in fruits, vegetables, tea, and plant-derived foods—as adjunctive agents in depression treatment [18]. For instance, quercetin and epigallocatechin gallate, two extensively studied flavonoids, demonstrate antidepressant effects by suppressing pro-inflammatory cytokines and enhancing BDNF expression in preclinical models [40]. Notably, a recent clinical review emphasized the ability of the flavonoids to modulate the gut–brain axis via microbiota composition regulation, thereby improving depressive symptoms through the serotonergic and vagal pathways [41]. In addition, polyphenols such as resveratrol and curcumin show efficacy in mitigating hippocampal neurodegeneration and HPA-axis dysregulation [42,43], which are important features of chronic stress-induced depression. These findings highlight the therapeutic potential of flavonoid- and polyphenol-rich diets for depression; however, translational challenges such as bioavailability and interindividual variability exist. Future trials should standardize dietary protocols or use nutraceuticals to provide evidence-based recommendations. Overall, compared with traditional treatments, dietary flavonoids and polyphenols offer novel insights into depression management. SS, a natural flavonoid extracted from various traditional Chinese medicinal herbs, has anti-inflammatory, antioxidant, and anti-tumor properties [44]. Many flavonoid compounds possess antidepressant effects. For example, baicalin, a flavonoid derived from the Scutellaria root, improves depression-like behaviors in rats subjected to a chronic mild stress scenario [45,46]. Quercetin, a type of flavonoid found in apples, onions, and berries, alleviates depression-like behaviors in LPS-treated rats [47]. Furthermore, flavonoid intake can significantly alleviate depressive symptoms, demonstrating its beneficial effects in patients with depression [40]. Future high-quality clinical studies are warranted to validate the safety and effectiveness of flavonoids in the treatment of depression [48]. Several studies have demonstrated that SS reduces neuroinflammation. In a murine model simulating Aspergillus fumigatus keratitis, SS downregulated the expression of Toll-like receptor 4 (TLR4) and myeloid differentiation primary response 88 (MyD88), thereby inhibiting the production of IL-1β, TNF-α, and IL-6. In addition, SS reduced the infiltration of neutrophils and myeloperoxidase activity, thereby alleviating the inflammatory response [49]. SS reduced TLR4/Myd88/Dynamin-related protein 1-related mitochondrial fission and inhibited the mRNA and protein expressions of IL-1β, TNF-α, and IL-6 in oxygen-glucose deprivation-stimulated BV2 microglia cells [50]. In an LPS-induced microglial cell model, derivatives of SS, such as isoschaftoside, inhibited the pro-inflammatory factor levels by suppressing hypoxia-inducible factor-1 (HIF-1α)-mediated metabolic reprogramming [51]. Escin significantly improved depression-like behaviors in rats with CUMS-induced depression and reduced the hippocampal IL-1β, TNF-α, and IL-6 levels by regulating the brain-derived neurotrophic factor/tropomyosin receptor kinase B/cAMP response element-binding protein and TLR4/MyD88/NF-κB signaling pathways [52]. Honokiol alleviated depression-like behaviors in CUMS-induced rats by activating the HIF-1α-vascular endothelial growth factor (VEGF) signaling pathway [53]. These results imply that SS may exert antidepressant effects, and the mechanisms may involve inhibiting the TLR4/MyD88 pathway or activating the HIF-1α-VEGF signaling pathway to suppress inflammation. In the present study, SS ameliorated depression- and anxiety-like behaviors in the LPS-treated and CUMS model mice. Furthermore, the IL-1β, IL-6, and TNF-α levels in the hippocampal region and blood serum of the CUMS model mice were reduced by SS.

The hippocampus, a brain region vital for emotional regulation and cognitive function, often exhibits structural and functional abnormalities in individuals with depression, such as reduced volume and impaired neurogenesis, particularly in the dentate gyrus [54]. In a CUMS mouse model, microglia in the hippocampus were activated [55], releasing pro-inflammatory cytokines that damaged synaptic plasticity and neurogenesis in the hippocampus [56]. Neuroinflammation may disrupt neural circuitry and promote the development of depressive symptoms [57]. In a study using a CUMS model of depression, the TLR4/MyD88/NF-κB and HIF-1α-VEGF signaling pathways in the hippocampus underwent significant changes, contributing to neuroinflammation and depressive behaviors [52,53]. In further studies, changes in the TLR4/MyD88/NF-κB or HIF-1α-VEGF signaling pathways in the mouse hippocampus should be detected to explore the mechanisms underlying the antidepressant effects of SS.

This study had some limitations despite the compelling support for the antidepressant properties of SS. Given the potential confounding effects of estrous cycle-associated hormonal fluctuations on behavioral and molecular outcomes, we chose to use only male mice in this animal study. Consequently, the conclusions of this study may be relevant solely to male mice. The mechanisms behind the antidepressant effects of SS warrant further investigation. However, our study primarily used animal models, and additional clinical studies need to be conducted to validate the effectiveness and safety of SS.

## 5. Conclusions

In conclusion, we discovered that SS exhibits antidepressant and anxiolytic effects that may be associated with the normalization of pro-inflammatory cytokine levels under CUMS. Therefore, SS could be a viable and potential therapeutic option for depression. Further studies are required to clarify its efficacy.

## Data Availability

The data presented in this study are available on request from the corresponding author upon reasonable request.
